# Comparing Transcutaneous Electrical Nerve Stimulation Added to Thoracolumbosacral Orthosis with Thoracolumbosacral Orthosis Alone on Pain, Trunk Control, and Respiratory Function in Chronic Incomplete Spinal Cord Injury: A Pilot Randomized Controlled Trial

**DOI:** 10.3390/bioengineering13080925

**Published:** 2026-08-14

**Authors:** Hanieh Khaliliyan, Arash Sharafatvaziri, Mahmood Bahramizadeh, Amirhossein Zare, Majid Ansari, Saeed Reza Mehrpour, Mohammad Hossein Nabian, Mohamad Naghi Tahmasebi, Morteza Faghih Jouibari, Morad Karimpour, Ghazaleh Moradkhani, Kavita Batra

**Affiliations:** 1 Department of Orthotics and Prosthetics, School of Rehabilitation Sciences, Isfahan University of Medical Sciences, Isfahan 8174673461, Iran; haniehkhaliliyan@yahoo.com (H.K.); zareamirhossein75@yahoo.com (A.Z.); 2 Center for Orthopedic Trans-Disciplinary Applied Research, Tehran University of Medical Sciences, Tehran 1461884513, Iran; arash.sharafatvaziri@gmail.com (A.S.); mehrpour_saeed@yahoo.com (S.R.M.); dr.nabian@gmail.com (M.H.N.); mntahmasebi@gmail.com (M.N.T.); 3 Shefa Neuroscience Research Center, Khatam Alanbia Hospital, Tehran 145414515, Iran; 4 Neuromusculoskeletal Rehabilitation Research Center, University of Social Welfare and Rehabilitation Sciences, Tehran 1461884513, Iran; 5 Sports Medicine Research Center, Neuroscience Institute, Tehran University of Medical Sciences, Tehran 1461884513, Iran; majid.ansari@gmail.com; 6 Department of Neurosurgery, Shariati Hospital, Tehran University of Medical Sciences, Tehran 1411713135, Iran; mortezafaghihj@yahoo.com; 7 School of Mechanical Engineering, College of Engineering, University of Tehran, Tehran 1439957131, Iran; m.karimpour@ut.ac.ir (M.K.); ghazalemoradkhani@ut.ac.ir (G.M.); 8 Department of Medical Education and Office of Research, Kirk Kerkorian School of Medicine at UNLV, University of Nevada, Las Vegas, NV 89106, USA; kavita.batra@unlv.edu

**Keywords:** chronic incomplete spinal cord injury, transcutaneous electrical nerve stimulation, thoracolumbosacral orthosis, trunk control, chronic pain, respiratory function

## Abstract

**Background:** Individuals with spinal cord injury (SCI) frequently experience chronic pain, trunk instability, and respiratory dysfunction. This pilot trial investigated the synergistic effects of a combined neuro-orthotic approach (thoracolumbosacral orthosis (TLSO) plus transcutaneous electrical nerve stimulation (TENS)) versus TLSO alone on pain, trunk control, and respiratory function in chronic incomplete SCI. **Methods:** Twelve adults with chronic incomplete SCI (T6–T12) were randomized to a combined group (TLSO + TENS) or a control group (TLSO alone) for a four-week intervention. Outcomes included pain (Visual Analogue Scale), trunk control (Trunk Assessment Scale), and respiratory function (tidal volume [VT], respiratory rate [RR], oxygen uptake [VO_2_], ventilatory equivalent for oxygen [VE/VO_2_], and inspiratory duty cycle [Ti/Ttot]) assessed at three time points (before intervention, immediately after intervention, and after one month). **Results:** The combined group demonstrated a significant reduction in pain (47.8% vs. 10.4%, *p* = 0.01) and an improvement in Ti/Ttot (33.3% vs. 4.9%, *p* = 0.008) compared to the control group. Both groups showed significant improvements in VT and RR over time (*p* < 0.05), reflecting the biomechanical benefits of TLSO. **Conclusions:** Integrating TENS with TLSO offers a non-invasive strategy for managing chronic pain and improving respiratory pattern coordination in incomplete SCI. While TLSO optimizes basic ventilatory mechanics, adding TENS significantly enhances neural drive for efficient breathing and pain gating. Future large-scale trials incorporating active task-specific training are needed to restore functional trunk control.

## 1. Introduction

Spinal Cord Injury (SCI) is defined as structural and functional damage to the spinal cord resulting from traumatic events or non-traumatic factors [1]. Currently, approximately 15 million people are living with SCI [2]. The greatest increase in SCI cases is observed among older adults, largely due to the increasing incidence of osteoporosis and the increased risk of falls due to impaired balance in this population [3]. Statistics show that new cases of SCI worldwide vary from 250,000 to 500,000 per year [4,5]. SCI results in a rupture or abnormality in the ascending sensory tracts (spinothalamic and dorsal column pathways), descending motor tracts (corticospinal, reticulospinal, and vestibulospinal pathways), and descending autonomic pathways (sympathetic and parasympathetic fibers), thereby producing loss of sensation, voluntary movement, and autonomic regulation below the level of lesion [6]. The clinical manifestations are directly determined by the skeletal level of injury: more rostral lesions interrupt a greater number of spinal segments, resulting in more extensive functional deficits—for example, thoracic-level injuries (T6–T12) preserve upper-limb function but impair trunk stabilization, postural control, and respiratory mechanics, whereas cervical injuries additionally compromise upper-extremity function and diaphragmatic innervation [1,7,8,9,10]. Clinical studies have confirmed that a higher level of injury correlates with a worse prognosis and a greater likelihood of serious secondary consequences [7,8,9,10].

Chronic pain develops within months of both complete and partial spinal cord injuries. More than 80% of patients experience clinically significant pain [11], which they describe as burning, electric-like, and stabbing [12]. Post-SCI pain severely impairs sleep quality, mental health, activities of daily living (ADLs), and overall quality of life, leading to consequences beyond motor impairment [11,12]. Sensorimotor impairments affecting trunk, upper, and lower limb function can disrupt the synergy required for postural stability, increasing the risk of instability, falls, and fractures [13].

Sensorimotor impairments affecting trunk, upper, and lower limb function can disrupt the synergy required for postural stability, increasing the risk of instability, falls, and fractures [13]. In individuals with thoracic-level SCI, the upper limb musculature remains neurologically intact, as the cervical segments innervating the arms are spared above the level of injury. However, trunk postural control acts as a critical rate limiter on upper limb function. The impairment of trunk control is mainly due to dysfunction of the descending supraspinal pathways, including the corticospinal, reticulospinal, and vestibulospinal tracts, which results in the loss of voluntary motor commands and the regulation of postural reflexes [1]. This neurological deficit causes paralysis or paresis of the axial and proximal trunk muscles, leading to changes in the dynamic stiffness of the spine and the inability to generate the multiplanar torques necessary to stabilize the body’s center of mass [14].

The loss of proximal trunk stability disrupts anticipatory postural adjustments (APAs). In able-bodied individuals, the central nervous system proactively activates trunk muscles to predict and shift the center of pressure, pre-stabilizing the base of support before the onset of voluntary limb movements [13,14]. Following thoracic SCI, the abolition of these feedforward mechanisms means that the trunk cannot provide a stable mechanical anchor for the kinetic chain. As demonstrated by Desroches et al. [14], sensorimotor trunk impairments significantly alter upper limb joint kinematics and kinetics during functional activities (such as sitting pivot transfers); the neurologically intact upper limbs are forced to adopt compensatory, mechanically inefficient strategies to maintain balance. This compensatory demand severely constrains their capacity for force generation, reaching, and manipulation. Thus, despite preserved cervical innervation, the upper extremities remain functionally limited by the unstable postural platform upon which they must operate [13,14]. The central nervous system (CNS) simplifies the control of the trunk’ s many degrees of freedom by organizing muscles into coordinated activation groups, termed muscle synergies, which are recruited in specific temporal sequences to generate multiplanar postural torques efficiently [14]. Following SCI, the disruption of descending supraspinal drive and the loss of segmental afferent feedback impair both the recruitment and the temporal coordination of these synergies, resulting in abnormal co-activation patterns, reduced trunk mechanical efficiency, and a significant increase in energy expenditure during basic functions such as sitting and standing balance [13,14].

Trunk alignment and postural control significantly influence respiratory mechanics [15]. The trunk and abdominal muscles stabilize the thoracoabdominal system, which facilitates efficient diaphragmatic function and ventilation [16]. When trunk stability is compromised, respiratory mechanics can be altered, leading to changes in ventilatory patterns, reduced ventilatory efficiency, and increased work of breathing [15]. Consequently, individuals with SCI may experience cardiopulmonary limitations, such as altered oxygen consumption, ventilation, tidal volume, and breathing frequency, even at rest [17]. Restoring relaxed sitting and dynamic abilities offers clinical and physiological benefits by improving trunk posture and control. These benefits include reduced neck and back pain, enhanced ability to perform pressure relief, improved two-handed activities, better pelvic tilt, increased forward reaching and active lunging, and improved diaphragmatic and deep breathing [18,19].

Currently, three main types of treatment are used for this population: drug therapy, surgery, and rehabilitation [20]. However, conventional methods within these groups have significant drawbacks and are ineffective in controlling pain. The main goal of surgical treatment is to reduce pressure on the spinal cord and prevent secondary injury, but the repair of damaged nerve tissue remains controversial [21]. Drugs, such as hormones, are only effective in the early stages of injury and have limited long-term effects on nerve recovery [22].

Interventions on trunk function are commonly used in the rehabilitation of individuals with SCI [23]. It has also been reported that trunk function in individuals with SCI is related to sitting balance, walking ability, and activities of daily living [24]. Biomechanical device therapy with trunk orthoses in the form of thoracolumbosacral orthosis (TLSO) using the Three-Point Pressure System can lead to improved trunk alignment [25,26]. Some studies have reported that trunk orthoses have a small benefit for respiratory function. Subsequent studies have shown that abdominal binding techniques improve lung volume when the patient is sitting [27]. The primary goal of the TLSO was to support the trunk and the paralyzed or weak muscles, which can improve diaphragmatic function by expanding the diaphragm in the zone of opposition [28]. A decrease in abdominal compliance can have two contrasting effects. By increasing diaphragmatic function, inspiratory pressure and volume displacement are improved. This correction can lead to a decrease in breathlessness. However, a decrease in abdominal compliance can increase diaphragmatic load, which can have the opposite effect and increase breathing difficulty [27]. This investigation focuses on people whose neurological injury level lies between T6 and T12. This particular region is important from a mechanistic point of view since it involves the segmental innervation of the main trunk flexor and rotator muscles, such as the rectus abdominis, external oblique, internal oblique, and transversus abdominis muscles (T7–T12). The neurological lesion in this region affects the abdominal muscles but does not affect the upper extremity and diaphragm. Thus, the loss of these muscles leads to an inability to create IAP and thus prevents the mentioned synergism between breathing and posture, making the pelvis-lumbar region unstable.

Recent advances in neurorehabilitation have highlighted the capacity of spinal neural circuits below the level of injury to retain functional potential when stimulated [29]. Neuroregulation strategies aimed at modulating the excitability of spinal networks have demonstrated effects on motor control, posture, and locomotor function [30]. Electrical stimulation techniques have been explored as non-invasive approaches capable of enhancing the functional state of spinal circuitry by facilitating sensorimotor integration and promoting the activation of residual neural pathways [29,30,31]. These approaches may influence not only limb movement but also trunk stabilization and postural regulation, as postural and locomotor networks share common afferent inputs from visual, vestibular, and proprioceptive systems [29].

The currently available treatments include pharmacological, surgical, and rehabilitation modalities; however, these treatment options lack effective and sustained results in terms of pain reduction and functional improvement [20,21,22]. Trunk braces or orthoses, such as a TLSO, may enhance alignment of the trunk and also aid in mechanical functioning of respiration through the thoracoabdominal area [23,24,25,26,27,28]. Non-invasive neuromodulatory treatment methods, such as transcutaneous electrical nerve stimulation (TENS), may modulate spinal sensorimotor systems and reduce pain [29,30,31,32,33]. This dermatomal targeting was designed to primarily stimulate large-diameter cutaneous and peripheral Aβ afferents. Rather than directly modulating deep spinal motor networks, this sensory input facilitates segmentary pain gating and enhances cutaneous sensory feedback from the trunk. This augmented afferent input is hypothesized to indirectly support trunk stabilization and respiratory coordination by alleviating pain-induced motor inhibition and improving the central processing of trunk proprioception [32].

Thus, the primary objective of this study was the comparison of the effect of TLSO plus TENS to TLSO only on the level of pain intensity and trunk control. The secondary goals were to analyze the impact of this combination of treatments on respiratory functions, such as tidal volume, breathing frequency, oxygen consumption, ventilatory equivalent for oxygen, and inspiratory duty cycle. It is expected that the combination will show better results in terms of improvement in pain and trunk control and better change in the breathing pattern.

## 2. Materials and Methods

### 2.1. Trial Design and Setting

This pilot, repeated-measures, two-arm parallel-group randomized trial was conducted in accordance with the CONSORT 2025 statement [34] and approved by the Ethics Committee of Tehran University of Medical Sciences (Ethics Code: IR.TUMS.MEDICINE.REC.1404.377, received 21 September 2025). The trial was prospectively registered in the Iranian Registry of Clinical Trials on 3 October 2025 (IRCT: IRCT20240514061793N3). All clinical assessments were performed between 20 October 2025, and 26 May 2026. No methodological changes occurred after trial commencement. Participants were recruited from Rofeideh Rehabilitation Hospital, Tehran, Iran.

### 2.2. Participants

Potential participants were identified through a systematic review of medical records and clinical databases. Following an initial electronic screening to verify primary neurological and age eligibility, candidates were contacted via telephone to assess their interest and preliminary suitability.

Inclusion criteria comprised adults aged 18 to 65 years with a confirmed diagnosis of chronic incomplete SCI, defined as ≥6 months post-injury, with neurological levels ranging from T6 to T12. Both traumatic and non-traumatic etiologies of SCI were eligible for inclusion. Eligible individuals were required to demonstrate clinically significant deficits in trunk control or seated balance, defined as the ability to maintain an unsupported sitting posture for at least 30 s while exhibiting instability during dynamic reaching tasks [33]. Furthermore, participants had to report persistent, non-acute SCI-related pain lasting for a minimum of three months prior to enrollment, with a baseline pain intensity score of ≥30 mm on the Visual Analogue Scale (VAS) to ensure clinical relevance [32]. Participants taking maintenance medications for spasticity (e.g., baclofen, tizanidine) or chronic pain were permitted to enroll provided their pharmacological regimen had been stable for a minimum of three months prior to baseline assessment and remained unchanged throughout the trial duration. All participants were medically stable, free from recent acute exacerbations, possessed sufficient cognitive function to comprehend the experimental protocols, and were physically capable of tolerating the TLSO, TENS electrodes, and the respiratory assessment facemask.

Exclusion criteria encompassed a history of spinal surgery within the preceding six months or severe spasticity (Modified Ashworth Scale > 3) that could interfere with orthotic fitting, electrode placement, or assessment protocols. Patients presenting with active pressure ulcers, skin breakdown, or dermatological conditions in areas designated for TLSO contact or TENS electrode placement were excluded. Additional exclusion criteria included pregnancy or lactation, severe, uncontrolled cardiopulmonary diseases, co-occurring neurological disorders affecting trunk musculature or balance, the use of concurrent neuromodulation therapies, and contraindications to electrical stimulation such as implanted cardiac pacemakers, metallic implants in the stimulation area, or severe sensory hypersensitivity [26].

### 2.3. Interventions

Two primary interventions were implemented in the present study. Participants were allocated to either the combined intervention group or the control group. The combined intervention group received a TLSO + TENS, while the control group received TLSO. We designed this combined neuro-orthotic intervention because we aimed to utilize both interventions concurrently in the trial group.

The TLSO used in this study was a prefabricated brace (TanTeb, Tehran, Iran) that was fitted by an orthotist for each participant. The brace’s trimlines were customized to optimize fit, stability, and individual comfort. The anterior trimline extended superiorly to just below the costal margin and inferiorly to the symphysis pubis. The posterior trimline spanned from the level of the scapular spines superiorly down to the gluteal crease inferiorly. The lateral trimlines were customized to cover the flank region, extending from the inferior costal margin (specifically below the 11th and 12th ribs) superiorly to just above the iliac crest inferiorly. In the anterior–posterior dimension, the lateral straps extended from the anterior axillary line to the posterior axillary line. This anatomical contouring was designed to prevent rib compression and avoid restricting diaphragmatic excursion, while simultaneously providing optimal lumbopelvic stability and preserving hip joint mobility [26]. The orthosis featured semi-rigid plastic panels with foam padding, and all trimlines were fine-tuned by the orthotist in both sitting and standing positions to ensure optimal trunk support, minimize pressure points, and maintain skin integrity.

**Figure 1:**
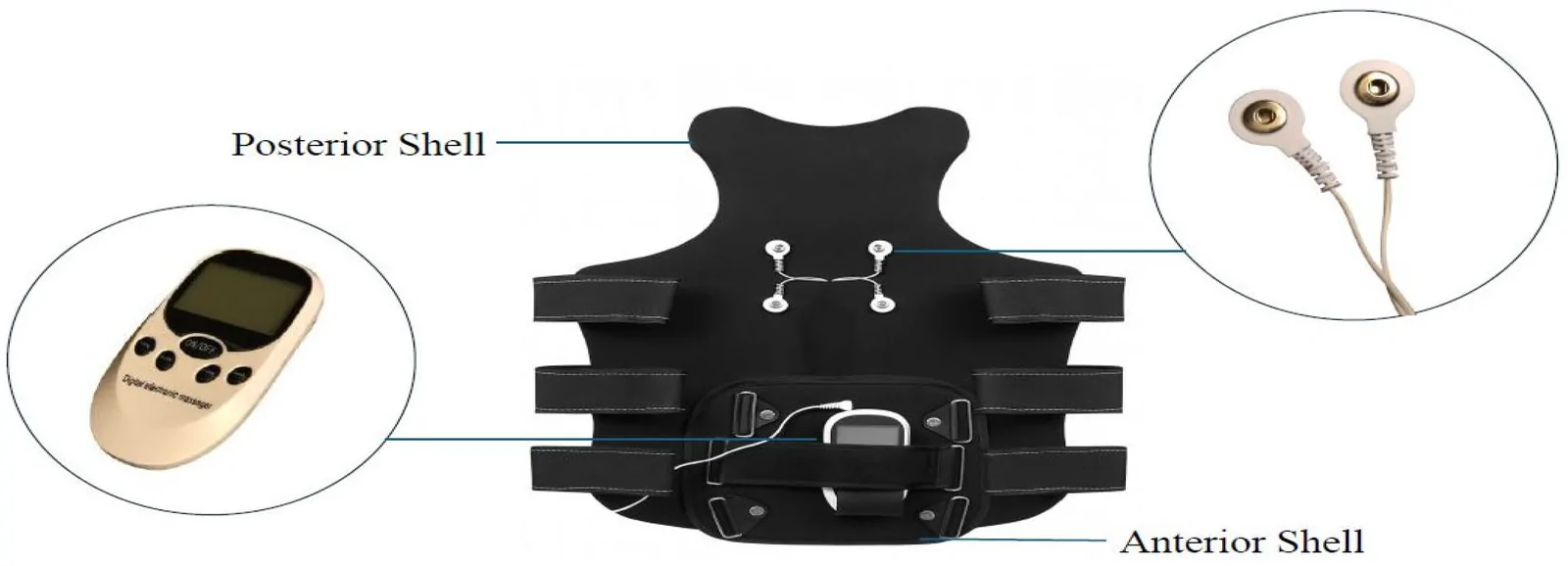
The combined neuro-orthotic intervention, showing the prefabricated thoracolumbosacral orthosis (TLSO) with semi-rigid plastic panels and customized trimlines, the portable dual-channel TENS device (Carina, model XTK-058) with four self-adhesive surface electrodes, and electrode placement over the thoracolumbar paraspinal muscles, 2–3 cm lateral to the spinous processes at the level of injury.

In the combined intervention group, TENS was administered using a portable dual-channel TENS device (Carina, model XTK-058, Suzhou, China), featuring four self-adhesive surface electrodes connected to two independent output channels. For the purpose of this study, a high-frequency TENS protocol was employed to optimize both analgesic and neuromodulatory effects. The device was set to a frequency of 80–100 Hz with a pulse width of 200 μs [32,33]. Electrode placement was individualized based on each participant’s neurological level of injury. The active electrodes were positioned bilaterally over the paraspinal muscles, centered 2–3 cm lateral to the spinous processes at the injured spinal segments [23]. This dermatomal and myotomal targeting was designed to directly stimulate the dorsal root afferents and spinal interneuronal networks at the level of the lesion, facilitating sensory integration for pain gating and enhancing the excitability of residual thoracolumbar circuits involved in trunk stabilization and respiratory motor coordination [33]. The stimulation intensity was individually titrated to the maximum tolerable sensory level (strong paresthesia) below the motor threshold to avoid any visible muscle contractions or spasms, ensuring selective activation of large-diameter afferent fibers (Aβ) for neuromodulation [31]. Participants in both groups were instructed to wear the TLSO for approximately 6–8 h per day during their usual daily activities. In the combined intervention group, TENS was additionally applied once daily for 20 min (30 sessions during one-month follow-up) [32,33]. The combined intervention is illustrated in Figure 1.

### 2.4. Outcomes

In this study, pain, and trunk control parameters were defined as the primary outcomes, while respiratory parameters were considered as the secondary outcomes. All assessments were conducted in a musculoskeletal laboratory environment (temperature ≈ 22 °C, illumination 120 lux, relative humidity 15%, and ambient noise 40 dB). Measurements were performed at three time points: baseline (before any intervention), immediately after intervention (after 20 min of adaptation), and after one month of intervention use.

#### 2.4.1. Pain

Pain intensity was assessed using the Visual Analogue Scale (VAS). The VAS consisted of a 10 cm horizontal line without numerical markings, with the left end representing “no pain” and the right end representing “worst imaginable pain.” Participants were instructed to mark on the line the point that best represented the intensity of their current pain. The distance from the left anchor to the participant’s mark was then measured in centimeters using a ruler, providing a quantitative score ranging from 0 to 10 cm. In individuals with SCI, the VAS has demonstrated excellent test–retest reliability (ICC = 0.83–0.96) and strong concurrent validity (correlation coefficients of 0.72–0.92) [35].

#### 2.4.2. Trunk Control

Trunk control was evaluated using the Trunk Assessment Scale for individuals with Spinal Cord Injury (TASS). The TASS is a functional assessment tool composed of nine tasks designed to examine trunk performance in individuals with SCI [36]. Among these tasks, one evaluates static sitting stability, while the remaining eight assess dynamic trunk movements. The structure and scoring of the scale were originally developed through a Delphi consensus process involving experienced physical therapists specialized in the rehabilitation of individuals with SCI [37]. Each task is scored on a graded scale ranging from 0–2, 0–4, or 0–6, depending on the complexity of the movement, yielding a maximum total score of 44, with higher scores reflecting better trunk control.

**Figure 2:**
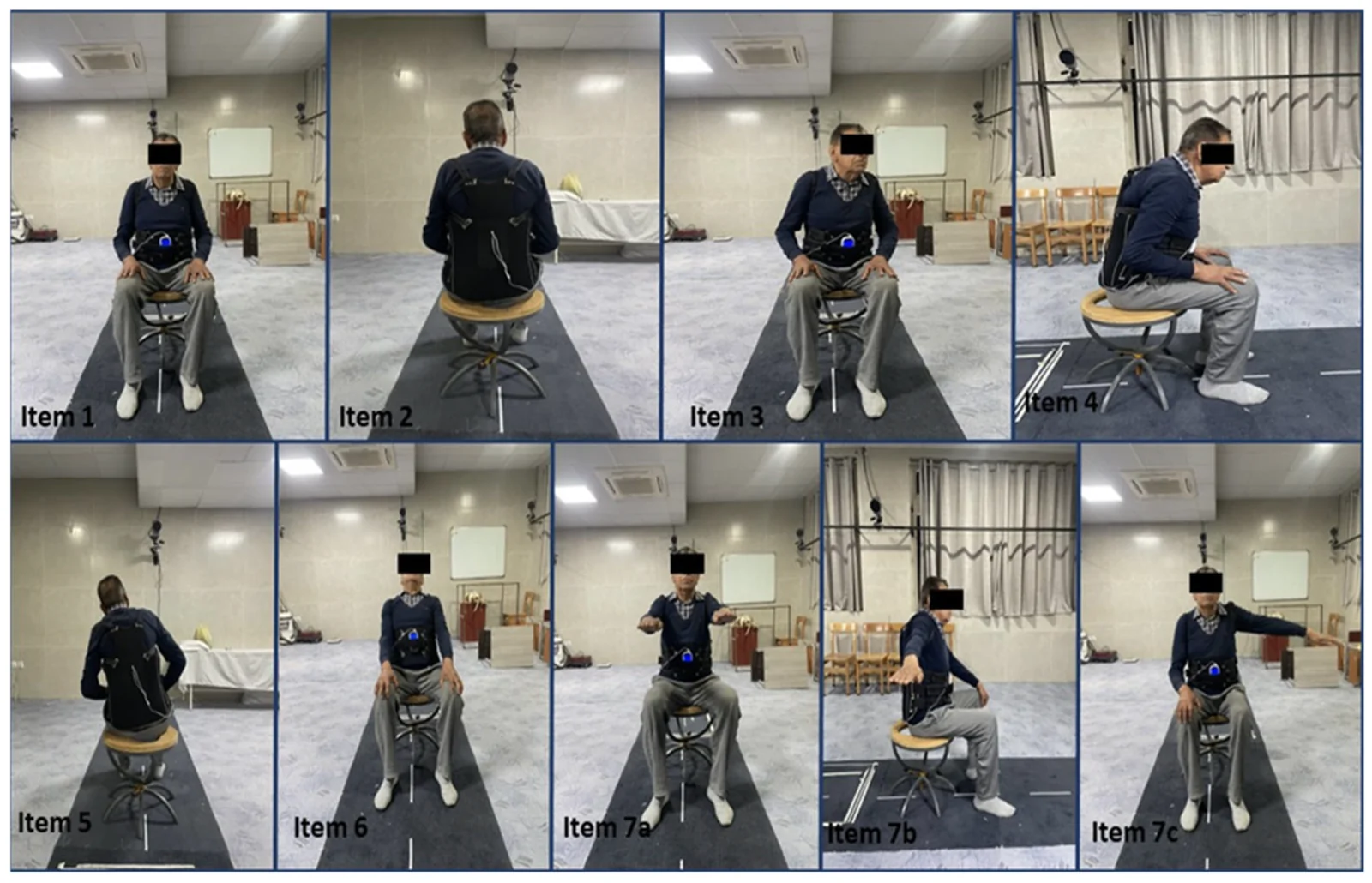
A participant performing selected items of the Trunk Assessment Scale for Individuals with Spinal Cord Injury (TASS). The participant is seated on a firm, flat chair without a backrest, with hips and knees at approximately 90° of flexion. The images show unsupported sitting posture, forward trunk flexion, lateral trunk flexion, trunk rotation, and anterior reaching tasks. No upper-extremity support or lower-limb orthoses were permitted during testing.

During the assessment, participants were seated on a firm, flat chair without a backrest, with the hips and knees positioned at approximately 90° of flexion. Participants were permitted to wear their regular footwear, but the use of lower-limb orthoses was not allowed. In addition, support with the upper extremities was not permitted during testing to ensure that trunk performance was assessed independently. The evaluation included the following tasks: maintenance of unsupported sitting posture, unilateral elevation of the ischial tuberosity, trunk rotation, forward trunk flexion, lateral trunk flexion, posterior trunk tilt, and reaching tasks in anterior, right, and left directions. The reaching component required participants to reach in three directions on each side, and incorporates procedures comparable to the Seated Reach Test and the Shoulder Thrust Test, which have been reported to be compatible with this item of the scale [36]. The final score obtained from all items was used to represent the overall level of trunk control (Figure 2). The TASS was administered and scored by an independent examiner who was blinded to treatment group allocation and was not involved in intervention delivery or randomization.

#### 2.4.3. Respiratory Function

**Figure 3:**
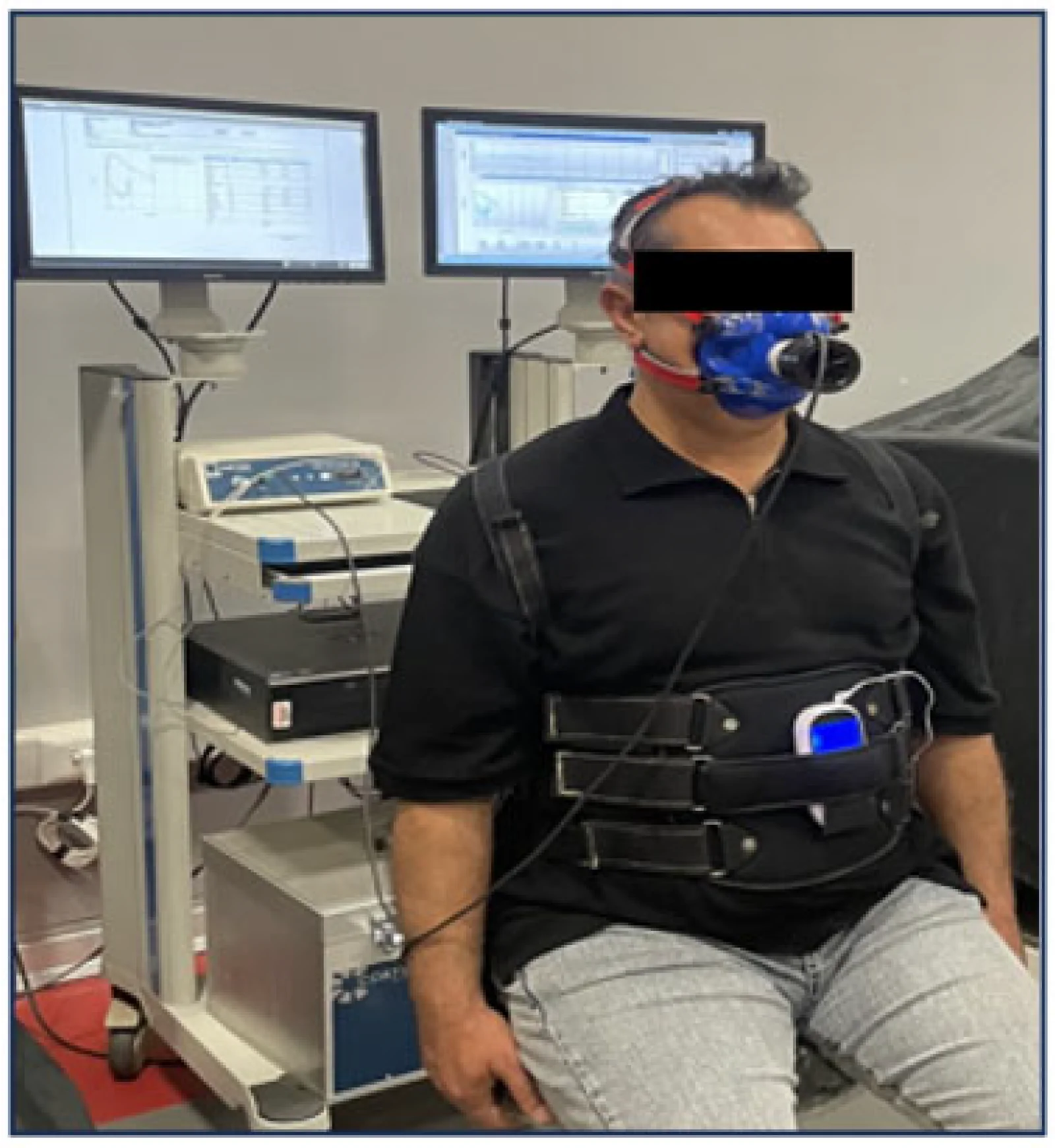
Respiratory gas exchange assessment using the MetaLyzer 3B metabolic analysis system (CORTEX Biophysik GmbH, Leipzig, Germany).

Respiratory gas exchange parameters were assessed using a metabolic gas analysis system (MetaLyzer 3B, CORTEX Biophysik GmbH, Leipzig, Germany) (Figure 3). The system operates on a breath-by-breath analysis principle, enabling continuous measurement of ventilatory and metabolic variables at rest. Prior to each assessment session, the device was calibrated according to the manufacturer’s guidelines, including gas calibration with reference gases and volume calibration using a 3 L syringe to ensure measurement accuracy. During testing, participants were seated comfortably in a standardized upright posture while wearing their assigned intervention. A fitted facemask connected to the metabolic cart was used to collect expired gases, and a nose clip was applied to prevent nasal airflow leakage. Participants were instructed to breathe normally and continuously throughout the measurement period while minimizing head movement and avoiding speech to ensure accurate breath-by-breath recording. The protocol included a continuous resting measurement phase lasting 5 min. To account for initial reactivity to the facemask and equipment, the first 2 min were considered an adaptation period, and the steady-state respiratory variables recorded during the final 3 min were averaged for statistical analysis [15]. Outcome measures related to respiratory function included oxygen uptake (VO_2_), minute ventilation (VE), tidal volume (VT), respiratory rate (RR), ventilatory equivalent for oxygen (VE/VO_2_), and inspiratory duty cycle (Ti/Ttot). These parameters were obtained directly from the breath-by-breath gas analysis system and used to characterize respiratory and metabolic responses during the testing protocol [16].

### 2.5. Sample Size

An a priori sample size estimation was conducted using G*Power software (Version 3.1, University of Düsseldorf, Germany). Based on the study design, the test family was defined as F tests, and the statistical test was specified as ANOVA: Repeated measures, within-between interaction. Drawing on findings from a previous study that reported a Cohen’s d effect size of 0.46 for changes in the VAS score following a TENS intervention [38], and considering a statistical power of 0.60, representing the minimum acceptable level for pilot studies, and a significance level of α = 0.10 to account for constraints in participant availability [39], the required sample size was determined. Additional input parameters included two measurement groups and three repeated measurements (baseline, post-intervention, and one-month follow-up), with an assumed correlation among repeated measures of 0.50 and a nonsphericity correction (ε) of 1. The analysis indicated a required total sample size of 12 participants, resulting in an allocation of 6 participants per group.

### 2.6. Randomization

The random allocation sequence for this two-arm parallel randomized controlled trial was generated using the online randomization service available at Randomization.org. Block randomization with a fixed block size of four was employed. In addition, the order of the assessment tests was randomized for each participant using the same online platform to minimize potential order effects, including fatigue and learning bias. The allocation sequence was implemented using sequentially numbered, opaque, sealed envelopes prepared by an independent researcher who was not involved in participant recruitment or outcome assessment. Each envelope contained the pre-generated group assignment according to the block randomization schedule. Envelopes were opened sequentially only after completion of baseline assessments, thereby ensuring allocation concealment until assignment. An independent investigator generated the block randomization sequence via Randomization.org and prepared the sealed envelopes. A separate researcher enrolled participants and assigned them to the intervention groups by opening the next envelope in numerical order. Outcome assessors were not involved in sequence generation or allocation procedures. Due to the nature of the intervention, participant blinding was not feasible.

### 2.7. Adherence, Concomitant Care, and Harms

To monitor participant compliance, participants were provided with a structured daily compliance checklist to record the duration of daily TLSO wear, the frequency and duration of TENS sessions, and any deviations from the protocol. Furthermore, a research assistant conducted telephone follow-ups on a weekly basis. These weekly calls were utilized to verify the log entries, calculate overall compliance rates, troubleshoot practical barriers to device usage, and reinforce adherence to the prescribed protocols.

To prevent potential disuse atrophy, core muscle deconditioning, or joint stiffness associated with daily TLSO use, a core maintenance and mobility protocol was prescribed to both study groups. This protocol consisted of a 15 min daily routine performed without the TLSO. The exercises included: (1) seated anterior and posterior pelvic tilts to maintain lumbopelvic mobility; (2) submaximal isometric abdominal bracing and gentle, pain-free trunk rotations to preserve core muscle tone; and (3) active upper-extremity reaching and stretching tasks to promote overall postural mobility [40].

During the weekly telephone inquiries and in-person follow-up assessments, participants were evaluated for intervention-related complications. Inquiries focused on skin integrity (e.g., erythema, pressure ulcers, or contact dermatitis beneath the TLSO or TENS electrodes), exacerbation of neuropathic pain, increased spasticity, or any unusual autonomic symptoms. All participants were instructed to immediately report any discomfort or unusual symptoms. All reported adverse events were documented, graded for severity, and managed according to standard clinical guidelines. No severe adverse events directly related to the interventions were reported during the trial. Adherence was monitored as a feasibility indicator and summarized descriptively.

### 2.8. Statistical Methods

All statistical analyses were performed using SPSS (version 25; IBM Corp., Armonk, NY, USA). The normality of continuous variables was assessed using the Shapiro–Wilk test. In addition, normal distribution was visually inspected using Q–Q plots and histograms. Baseline demographic and clinical characteristics were compared between the two groups to ensure initial comparability. For continuous variables, independent samples t-tests were used when normality assumptions were satisfied. For categorical (nominal) variables, Chi-square (χ^2^) tests of independence were performed. In cases where expected cell counts were less than five, Fisher’s exact test was applied.

Continuous outcome variables derived from repeated measurements were analyzed using a repeated measures analysis of variance (RM ANOVA), with Time specified as the within-subject factor and Group (intervention vs. control) as the between-subject factor. The model evaluated the main effects of Time, Group, and Group × Time interaction. The Group × Time interaction was considered the primary effect of interest, as it reflects differential changes in outcomes between groups across the measurement time points and thus indicates the presence of a treatment effect over time. At this stage, *p* < 0.05 was considered significant.

Assumptions for the RM ANOVA were evaluated. Homogeneity of variances between groups was examined at each measurement point using Levene’s test. For within-subject effects comprising more than two time points, the sphericity assumption was tested using Mauchly’s test of sphericity. In cases where this assumption was violated (*p* < 0.05), the Greenhouse-Geisser correction was applied to adjust the degrees of freedom and provide valid F-statistics. When significant main effects or interaction effects were detected in the RM ANOVA, post hoc pairwise comparisons were performed to further explore the nature of the differences. These comparisons included within-group analyses across different time points as well as between-group comparisons at each measurement time point. To control for the inflation of Type I error associated with multiple comparisons, Bonferroni correction was applied to adjust the significance levels of pairwise tests [41]. In this stage, *p* ˂ 0.006 was considered significant.

To quantify the magnitude of the observed effects, effect size measures were calculated and reported. For the RM ANOVA, partial eta squared (η^2^) was used to estimate the effect size for the main effects of Time, Group, and the Group × Time interaction. The magnitude of η^2^ was interpreted according to thresholds, with values of 0.01, 0.06, and 0.14 representing small, medium, and large effects, respectively. For pairwise comparisons, Cohen’s d was calculated to estimate standardized mean differences. Cohen’s d values of 0.20, 0.50, and 0.80 were interpreted as small, medium, and large effects, respectively. For between-group comparisons, Cohen’s d was computed using the pooled standard deviation of the two groups, whereas for within-group comparisons over time, the standardized mean change approach was applied. Given the exploratory nature of this pilot randomized controlled trial, emphasis was placed not only on statistical significance but also on the magnitude and precision of treatment effects, as reflected by effect size estimates and confidence intervals [41].

## 3. Results

### 3.1. Baseline Data and Participants’ Flow

**Figure 4:**
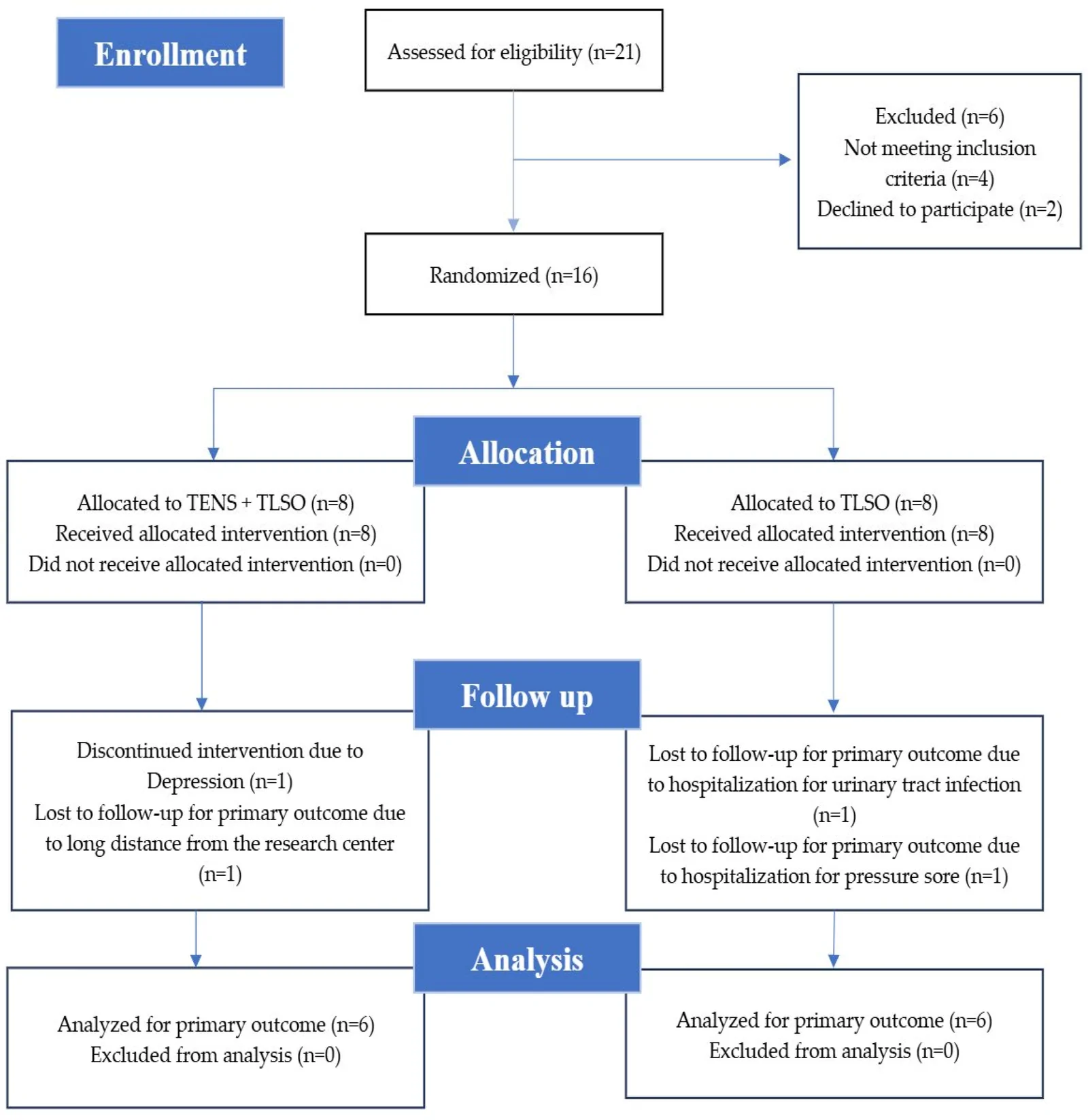
CONSORT 2025 participant flow diagram.

Table 1 summarizes the demographic and clinical characteristics of participants at baseline. There were no significant differences between groups in any baseline variables. Figure 4 presents the CONSORT flow diagram illustrating participant progression through the trial. Of the 21 individuals with SCI assessed for eligibility, 12 participants (6 in each group) finally completed the three assessment sessions, and their data were analyzed.

### 3.2. Primary and Secondary Outcomes

Table 2 presents the results of RM ANOVA for all outcome variables across the three assessment time points. A significant group × time interaction was observed for pain intensity (Wilk’s Lambda = 0.39, F (2, 9) = 6.85, *p* = 0.01), with a large effect size (ŋ2 = 0.60), indicating that 60% of the variance in pain reduction was attributable to the intervention. The TLSO + TENS group demonstrated a 47.8% reduction in pain scores from baseline (4.31 ± 1.51 cm) to final follow-up (2.25 ± 0.76 cm), whereas the TLSO-only group showed a modest 10.4% reduction (4.33 ± 1.52 cm to 3.88 ± 1.54 cm). A significant main effect of time was also detected (ŋ2 = 0.24, F (2, 9) = 13.87, *p* = 0.002, ŋ2 = 0.75), indicating that pain scores changed across assessment points regardless of group allocation. No significant between-group differences were found (*p* = 0.37).

No significant group × time interaction emerged for trunk control scores (Wilk’s Lambda = 0.86, F (2, 9) = 0.67, *p* = 0.53), with a small effect size (ŋ2 = 0.13) suggesting that only 13% of variance was explained by the combined intervention. However, a significant main effect of time was observed (Wilk’s Lambda = 0.38, F (2, 9) = 7.08, *p* = 0.01, ŋ2= 0.61), indicating that trunk control improved over time in both groups, with a large effect size explaining 61% of the variance. Between-group differences were non-significant (*p* = 0.58, ŋ2 = 0.03).

For VT, no significant group × time interaction was detected (Wilk’s Lambda = 0.74, F (2, 9) = 1.54, *p* = 0.26), with a medium effect size (ŋ2 = 0.25). However, a highly significant main effect of time emerged (Wilk’s Lambda = 0.17, F (2, 9) = 21.24, *p* < 0.001), with a large effect size (ŋ2 = 0.82), indicating that 82% of the variance in VT was explained by time. The TLSO + TENS group showed an 8.3% increase in VT (419.5 mL to 454.33 mL), while the TLSO group demonstrated a 4.4% increase (424.5 mL to 443.00 mL). RR exhibited a non-significant group × time interaction (Wilk’s Lambda = 0.70, F (2, 9) = 1.88, *p* = 0.20, ŋ2 = 0.29), though the medium effect size suggests a trend. A significant main effect of time was detected (Wilk’s Lambda = 0.34, F (2, 9) = 8.37, *p* = 0.009, ŋ2 = 0.65), with both groups showing reductions: 17.1% decrease in the TLSO + TENS group (15.47 to 12.83 breaths/min) and 15.1% decrease in the TLSO group (15.07 to 12.80 breaths/min). The Ti/Ttot demonstrated a significant group × time interaction (Wilk’s Lambda = 0.34, F (2, 9) = 8.49, *p* = 0.008), with a large effect size (ŋ2 = 0.65) indicating that 65% of variance was explained by the intervention. The TLSO + TENS group showed a 33.3% increase in Ti/Ttot (0.39 to 0.52), compared to a modest 4.9% increase in the TLSO group (0.41 to 0.43). A significant main effect of time was also observed (Wilk’s Lambda = 0.26, F (2, 9) = 12.73, *p* = 0.002, ŋ2 = 0.73).

No significant effects were observed for VE, VO_2_, or VE/VO_2_, with all *p*-values > 0.05 and trivial to small effect sizes (ŋ2 ranging from 0.01 to 0.38), suggesting that these parameters were not influenced by either intervention.

### 3.3. Pairwise Comparisons and Estimates

At baseline (T1), no significant differences were observed between groups for any outcome measure, confirming successful randomization. At T2, between-group comparisons remained non-significant across all variables. However, at final follow-up (T3), significant between-group differences emerged for two primary outcomes. For pain intensity, the TLSO + TENS group demonstrated significantly lower VAS scores compared to the TLSO-only group at T3 (MD = −1.63 cm, 95% CI: −3.20 to −0.07, *p* = 0.04), with a large effect size (Cohen’s d = 1.34). This represents a difference exceeding the minimal clinically important difference (MCID) of 1.3 cm for pain in SCI populations [42]. Also, Ti/Ttot showed a significant between-group difference at T3 (MD = 0.09, 95% CI: 0.01 to 0.17, *p* = 0.03), with a large effect size (Cohen’ s d = 1.73). This indicates that participants receiving the combined intervention achieved a 21% higher Ti/Ttot ratio compared to those using TLSO alone.

The TLSO + TENS group demonstrated significant pain reduction across all time intervals. From baseline to T2, pain decreased by 0.60 cm (*p* = 0.004, d = 1.88), representing a 13.9% reduction. The reduction continued from baseline to T3, with a mean decrease of 1.25 cm (*p* = 0.001, d = 2.32), corresponding to a 29.0% reduction. The T2-to-T3 comparison also showed significant improvement (0.64 cm reduction, *p* = 0.004, d = 1.74), indicating sustained effects throughout the intervention period. All effect sizes were large (d > 1.5). Despite the significant main effect of time observed in RM ANOVA, pairwise comparisons within the TLSO + TENS group revealed no significant improvements in trunk control scores across any time interval (*p* values ranging from 0.07 to 1.00). The T1-to-T2 comparison showed a non-significant trend toward improvement (MD = 1.00, *p* = 0.07, d = 0.82), but this was not maintained at T3 (MD = 0.00, *p* = 1.00). TV demonstrated a significant 8.3% increase from baseline to T3 (MD = −34.83 mL, *p* < 0.001, d = 2.21), with large effect sizes indicating clinical improvement. The T2 to T3 comparison also reached significance (MD = −46.33 mL, *p* = 0.002, d = 1.73), suggesting progressive enhancement in inspiratory capacity. Respiratory rate showed a significant 17.1% reduction from baseline to T3 (MD = 2.64 breaths/min, *p* = 0.01, d = 1.31), with the most change occurring between T2 and T3 (3.49 breaths/min reduction, *p* = 0.004, d = 1.55). This pattern suggests a delayed but meaningful effect on breathing efficiency. The Ti/Ttot ratio exhibited the most improvement, with a 33.3% increase from baseline to T3 (MD = −0.12, *p* = 0.001, d = 2.45). The T2 to T3 comparison also showed significant enhancement (MD = −0.06, *p* < 0.001, d = 2.45).

The TLSO group showed minimal and nonsignificant changes in pain scores across all time intervals. The T1 to T2 comparison revealed a nonsignificant 0.36 cm reduction (*p* = 0.09, d = 0.77), representing only an 8.3% decrease. No further improvements were observed from T2 to T3 (*p* = 0.71). Unlike the combined intervention group, the TLSO group demonstrated improvements in trunk control from baseline to T2 (MD = 1.66, *p* = 0.008, d = 1.36), representing a 5.4% improvement. However, this gain was not sustained at T3, with scores returning to near-baseline levels (MD = −0.33, *p* = 0.41). The T2 to T3 comparison showed borderline significance (MD = −2.00, *p* = 0.006, d = −1.43). TV showed a trend to increase from baseline to T3 by 4.4% (MD = −18.50 mL, *p* = 0.01, d = 1.17). The T2-to-T3 comparison also showed significant trends (MD = −32.00 mL, *p* = 0.01, d = 1.18). RR decreased by 15.1% from baseline to T3 (MD = 2.26 breaths/min, *p* = 0.02, d = 1.13), with effect sizes in the large range. However, the T2-to-T3 comparison showed trends toward significance (*p* = 0.05, d = 0.90). The Ti/Ttot ratio showed no significant changes across any time interval in the TLSO group (*p* values ranging from 0.42 to 0.57), with trivial-to-small effect sizes (d < 0.41). This finding suggests that TLSO alone does not influence breathing pattern coordination.

**Figure 5:**
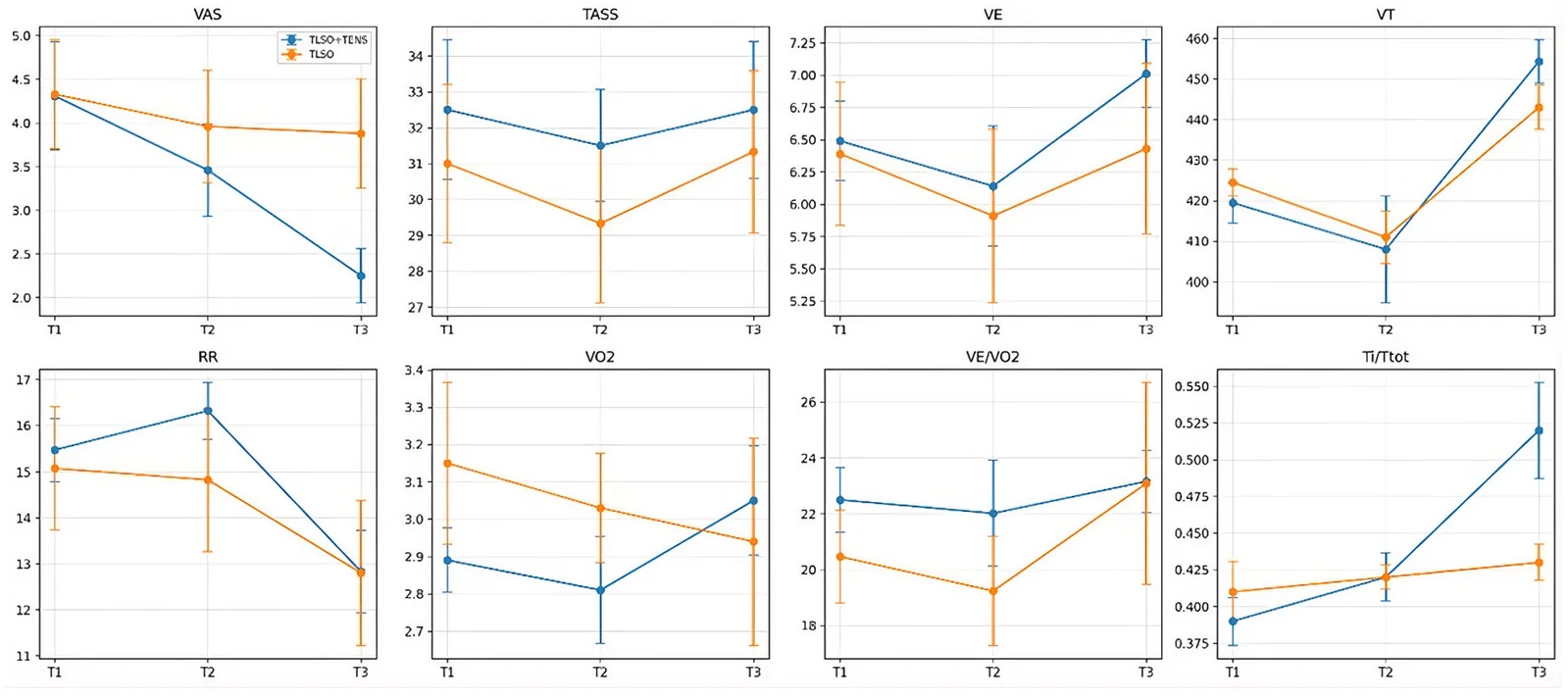
Longitudinal changes in primary and secondary outcomes across three assessment time points (T1: baseline; T2: immediately post-intervention; T3: one-month follow-up) in the TLSO + TENS group (blue) and the TLSO-only group (orange). Data are presented as mean ± standard deviation. The composite figure consists of eight panels arranged in two rows. The top row, from left to right, shows: pain intensity (VAS, cm); trunk control (TASS total score); minute ventilation (VE, L/min); and tidal volume (VT, mL). The bottom row, from left to right, shows: respiratory rate (RR, breaths/min); oxygen uptake (VO2, mL/min); ventilatory equivalent for oxygen (VE/VO2); and inspiratory duty cycle (Ti/Ttot). Asterisks indicate statistically significant Group × Time interactions (*p* < 0.05). TLSO: Thoracolumbosacral Orthosis; TENS: Transcutaneous Electrical Nerve Stimulation.

The TLSO + TENS group demonstrated large effect sizes (d > 1.30) for pain reduction, TV enhancement, respiratory rate reduction, and Ti/Ttot improvement, indicating treatment effects (Table 3). The TLSO group showed more modest effects, with large effect sizes for RR reduction and moderate effects for TV changes, but minimal impact on pain and breathing pattern efficiency. The changes in each group are shown in Figure 5.

### 3.4. Intervention Adherence

Adherence was monitored using daily compliance checklists and weekly telephone follow-ups; however, complete quantitative wear-time data were not available for all participants. Therefore, adherence could not be formally analyzed as a primary or secondary outcome and is acknowledged as a limitation of the present study.

## 4. Discussion

The present pilot randomized trial investigated the synergistic effects of a combined neuro-orthotic approach (TLSO + TENS) compared to orthotic support alone (TLSO) on pain, trunk control, and respiratory function in individuals with chronic incomplete SCI. The combined intervention resulted in a significant reduction in pain intensity and an improvement in Ti/Ttot. While both groups exhibited significant improvements in VT and RR over time, the enhancement in breathing pattern coordination (Ti/Ttot) was exclusive to the combined group. Furthermore, although a main effect of time was observed for trunk control, neither intervention produced sustained functional improvements in this domain over the one-month follow-up. These results highlight the distinct yet complementary roles of biomechanical stabilization and non-invasive neuromodulation in addressing the multifaceted deficits following SCI.

### 4.1. Neuromodulatory and Biomechanical Effects on Pain and Respiratory Function

Chronic pain is a pervasive complication following SCI, limiting physical function and increasing the risk of secondary complications. In our study, the TLSO + TENS group demonstrated a 47.8% reduction in pain, exceeding MCID [42], whereas the TLSO-only group showed negligible changes. This aligns with evidence supporting electrical stimulation as an effective neuromodulatory modality for managing chronic and neuropathic pain in SCI [32,33]. As outlined in the clinical guidelines for spinal cord stimulation, electrical stimulation modulates the sensory experience of pain by engaging spinal and supra-spinal inhibitory pathways, consistent with the gate control theory [43]. A systematic review by Khan et al. (2019) identified pain, alongside impaired balance and trunk weakness, as primary biological risk factors for falls and secondary musculoskeletal complications in the SCI population [44]. By significantly mitigating pain, the combined neuro-orthotic approach may not only improve the quality of life but also indirectly reduce fall risk and facilitate greater engagement in daily activities and active rehabilitation [11].

The improvement in ventilatory parameters, including VT and RR, across both groups over time can be attributed to the biomechanical effects of the TLSO. The mechanical support provided by the TLSO closely mirrors the effects of custom abdominal and truncal girdles described by Hart et al. (2005) [27]. In individuals with SCI, paralysis of the abdominal muscles leads to increased abdominal compliance and suboptimal operating lung volumes, which compromises diaphragmatic efficiency. Hart et al. demonstrated that truncal and abdominal support decreases dynamic abdominal compliance, reduces functional residual capacity, and increases inspiratory capacity. By optimizing the thoracoabdominal configuration and restituting the expanding effect of the diaphragm at the zone of apposition, the orthosis enhances diaphragm performance and reduces the sensation of respiratory effort [27]. Thus, the improvements in VT and RR observed in both study groups may be driven by the TLSO’s ability to optimize respiratory mechanics and reduce the work of breathing, independent of any neuromodulatory effects.

A 33.3% improvement in the Ti/Ttot ratio in the combined intervention group was observed. The Ti/Ttot ratio is an indicator of respiratory pattern coordination and the central neural drive to the respiratory muscles. It is crucial to distinguish the mechanisms of conventional TENS, as used in this study, from those of transcutaneous spinal cord stimulation (tSCS). While tSCS is specifically designed to recruit posterior root afferents to directly modulate spinal sensorimotor networks and facilitate motor output [45], conventional TENS primarily recruits large-diameter cutaneous and peripheral Aβ afferents. Therefore, direct extrapolation of tSCS mechanisms to conventional TENS requires caution and experimental validation. Instead, the improvement in Ti/Ttot observed with TENS is more plausibly explained by mechanisms consistent with conventional sensory-level stimulation: namely, altered sensory feedback, modulation of spinal reflex excitability, and indirect effects mediated through profound pain reduction.

Chronic neuropathic pain severely alters breathing patterns, often causing rapid, shallow breathing and restricting diaphragmatic excursion due to pain-induced splinting and inhibitory reflexes [17]. By effectively gating this pain via cutaneous Aβ afferent stimulation, TENS removes this inhibitory constraint, allowing for a more natural, coordinated inspiratory phase and an improved Ti/Ttot ratio. Furthermore, the continuous application of TENS over the thoracolumbar region provides augmented cutaneous sensory feedback. Cutaneous afferents play a vital role in postural awareness and sensorimotor integration. Enhancing this sensory input may improve the central processing of trunk position, thereby indirectly facilitating the coordination between postural and respiratory muscles [15]. This sensory and analgesic benefit represents a neurological advantage that purely biomechanical support, such as TLSO alone, cannot achieve.

### 4.2. Trunk Control and the Need for Active Training

Contrary to our initial hypothesis, the combined intervention did not produce sustained improvements in functional trunk control, as measured by the TASS. Both groups showed a transient main effect of time, but scores returned to baseline by the one-month follow-up. The TLSO provides external biomechanical stabilization, which limits excessive motion and may temporarily facilitate better postural alignment. However, long-term motor learning and neuroplastic reorganization of trunk musculature require active, task-specific engagement of the neural circuits [25,29]. As emphasized by Gad et al. (2018), while neuromodulation enables the spinal networks, the recovery of complex motor synergies is highly dependent on combining this enabling state with activity-dependent mechanisms, such as active motor training [45]. In the present study, TENS was applied passively at rest. Future protocols should investigate whether applying TENS concurrently with active, task-specific trunk training or weight-bearing activities could translate the enhanced neural excitability into sustained functional trunk control. Furthermore, Khan et al. (2019) noted that trunk weakness and impaired balance are multifactorial and heavily influenced by environmental and behavioral challenges; thus, improving TASS scores likely requires more intensive, ecologically valid training paradigms beyond passive orthotic and electrical support [44].

The rationale for combining TLSO and TENS stems from the understanding that SCI deficits are both biomechanical and neurological [9]. The findings of this pilot study suggest that these two modalities operate synergistically. The TLSO creates a stable mechanical environment and optimizes respiratory mechanics [27], potentially allowing the neuromodulatory effects of TENS to more effectively target the stabilization of respiratory patterns [45] and the alleviation of neuropathic pain [44,45] without the interference of compensatory movements.

### 4.3. Limitations

This study has several limitations that must be acknowledged. First, the TENS intervention was applied passively for a relatively short duration. As suggested by the neuromodulation literature, dose, duration, and the integration of active training are critical variables that dictate the extent of neuroplastic recovery [45]. Second, the lack of a sham-TENS control group means that the placebo effect cannot be entirely ruled out for the subjective pain outcomes, although the objective respiratory changes in Ti/Ttot strongly suggest a physiological mechanism. Third, the short follow-up period of one month may not have been sufficient to capture long-term neuroplastic adaptations in trunk control. Finally, this was a pilot study aimed at estimating effect sizes for future large-scale RCTs, and the power/alpha settings were chosen accordingly based on pilot study guidelines. Another limitation relates to intervention adherence. Although TLSO wear time and TENS session completion were monitored using self-reported diaries and weekly telephone follow-ups, adherence was not designated as a primary outcome and was not objectively measured using wearable adherence monitors.

Future research needs to concentrate on large-scale randomized clinical trials where the presence of a sham-TENS group would allow for a more accurate examination of the impact of neuromodulation as distinct from the placebo effect. In addition, future research needs to explore the long-term efficacy of the approach through the use of longer interventions and follow-ups to examine whether the adaptations in respiratory function and postural stability persist. Lastly, an essential next step would be the combination of the neuro-orthotic strategy with active trunk training.

## 5. Conclusions

The integration of TENS with TLSO offers a non-invasive strategy for managing chronic pain and improving respiratory pattern coordination in individuals with incomplete SCI. While biomechanical support alone aids in basic ventilatory mechanics by optimizing diaphragmatic function, the addition of neuromodulation is crucial for addressing neuropathic pain and enhancing the neural drive necessary for efficient breathing patterns. Future large-scale randomized controlled trials incorporating active, task-specific training alongside this neuro-orthotic approach are required to fully indicate its potential for restoring functional trunk control and mitigating fall risks in the SCI population.

## Figures and Tables

**Table 1:** The characteristics of participants.

Characteristics	TLSO + TENS M ± SD or No.	TLSO M ± SD or No.	*p* Value
Sex (F/M)	2/4	1/5	1
Age (years)	52.16 ± 6.45	50.83 ± 5.64	0.71
Height (cm)	169.83 ± 21.3	171.83 ± 19.60	0.86
Weight (kg)	81.33 ± 13.26	78.83 ± 5.67	0.68
Injury level	2T_7_/1T_9_/1T_10_/2T_11_	1T_7_/1T_8_/1T_10_/2T_11_/1T_12_	0.6
ASIA score	4C/2D	3C/3D	1
Etiology (Traumatic/Non-traumatic)	4/2	5/1	0.62
Duration since injury (mo)	16.21 ± 5.11	17.18 ± 4.31	0.73

ASIA: American Spinal Injury Association; M: Mean, SD: Standard Deviation.

**Table 2:** Descriptive statistics and results of repeated-measures ANOVA.

Outcome	Groups	Time			Group × Time	Time	Group
		T1 (M ± SD)	T2 (M ± SD)	T3 (M ± SD)			
VAS (cm)	TLSO + TENS	4.31 ± 1.51	3.46 ± 1.29	2.25 ± 0.76	Wilk’s Lambda = 0.39 F (2,9) = 6.85 *p* = 0.01 * ŋ2 = 0.6	Wilk’s Lambda = 0.24 F (2,9) =13.87 *p* = 0.002 * ŋ2 = 0.75	F (2,9) = 0.84 *p* = 0.37 ŋ2 = 0.7
	TLSO	4.33 ± 1.52	3.96 ± 1.57	3.88 ± 1.54			
TASS (total score)	TLSO + TENS	32.5 ± 4.76	31.5 ± 3.83	32.5 ± 4.67	Wilk’s Lambda = 0.86 F (2,9) = 0.67 *p* = 0.53 ŋ2 = 0.13	Wilk’s Lambda = 0.38 F (2,9) = 7.08 *p* = 0.01 * ŋ2 = 0.61	F (2,9) = 0.32 *p* = 0.58 ŋ2 = 0.03
	TLSO	31 ± 5.40	29.33 ± 5.42	31.33 ± 5.53			
VE (L/min)	TLSO + TENS	6.49 ± 0.75	6.14 ± 1.14	7.01 ± 0.64	Wilk’s Lambda = 0.84 F (2,9) = 0.8 *p* = 0.47 ŋ2 = 0.15	Wilk’s Lambda = 0.61 F (2,9) = 2.78 *p* = 0.11 ŋ2 = 0.38	F (2,9) = 0.19 *p* = 0.66 ŋ2 = 0.01
	TLSO	6.39 ± 1.36	5.91 ± 1.65	6.43 ± 1.62			
VT (mL)	TLSO + TENS	419.5 ± 12.14	408 ± 32.32	454.33 ± 12.95	Wilk’s Lambda = 0.74 F (2,9) = 1.54 *p* = 0.26 ŋ2 = 0.25	Wilk’s Lambda = 0.17 F (2,9) = 21.24 *p* ˂ 0.001 * ŋ2 = 0.82	F (2,9) = 0.03 *p* = 0.86 ŋ2 = 0.003
	TLSO	424.5 ± 8.09	411.00 ± 15.69	443.00 ± 13			
RR (breaths/min)	TLSO + TENS	15.47 ± 1.68	16.32 ± 1.51	12.83 ± 2.19	Wilk’s Lambda = 0.7 F (2,9) = 1.88 *p* = 0.2 ŋ2 = 0.29	Wilk’s Lambda = 0.34 F (2,9) = 8.37 *p* = 0.009 * ŋ2 = 0.65	F (2,9) = 0.17 *p* = 0.68 ŋ2 = 0.01
	TLSO	15.07 ± 3.27	14.82 ± 3.80	12.80 ± 3.84			
VO_2_ (mL/min)	TLSO + TENS	2.89 ± 0.21	2.81 ± 0.35	3.05 ± 0.36	Wilk’s Lambda = 0.69 F (2,9) = 1.94 *p* = 0.19 ŋ2 = 0.3	Wilk’s Lambda = 0.89 F (2,9) = 0.54 *p* = 0.59 ŋ2 = 0.1	F (2,9) = 0.31 *p* = 0.58 ŋ2 = 0.03
	TLSO	3.15 ± 0.53	3.03 ± 0.36	2.94 ± 0.68			
VE/VO_2_	TLSO + TENS	22.50 ± 2.86	22.02 ± 4.64	23.16 ± 2.72	Wilk’s Lambda = 0.92 F (2,9) = 0.35 *p* = 0.71 ŋ2 = 0.07	Wilk’s Lambda = 0.79 F (2,9) = 1.19 *p* = 0.34 ŋ2 = 0.21	F (2,9) = 0.42 *p* = 0.53 ŋ2 = 0.04
	TLSO	20.47 ± 4.08	19.24 ± 4.75	23.09 ± 8.85			
Ti/Ttot	TLSO + TENS	0.39 ± 0.04	0.42 ± 0.04	0.52 ± 0.08	Wilk’s Lambda = 0.34 F (2,9) = 8.49 *p* = 0.008 * ŋ2 = 0.65	Wilk’s Lambda = 0.26 F (2,9) =12.73 *p* = 0.002 * ŋ2 = 0.73	F (2,9) = 1.36 *p* = 0.27 ŋ2 = 0.12
	TLSO	0.41 ± 0.05	0.42 ± 0.02	0.43 ± 0.03			

Abbreviations: ANOVA: Analysis of Variance; VAS: Visual Analog Scale; TASS: Total Score; VE: Minute Ventilation; VT: Tidal Volume; RR: Respiratory Rate; VO2: Oxygen Consumption; VE/VO2: Ventilatory Equivalent for Oxygen; Ti/Ttot: Inspiratory Time Fraction (Duty Cycle); TLSO: Thoracolumbosacral Orthosis; TENS: Transcutaneous Electrical Nerve Stimulation; M: Mean; SD: Standard Deviation; T1, T2, T3: Time points; Wilk’s Lambda: Test statistic for repeated measures ANOVA; F: F-statistic; *p*: *p*-value; ŋ2: Partial Eta-squared (Effect size). * *p* < 0.05 was considered significant.

**Table 3:** Pairwise comparison.

Pairwise Between-Group Comparisons at Each Time Point				
Outcome	Time	Conditions	MD ± SE (95%CI)	*p* value (Cohen’s d)
VAS (cm)	T1	(TLSO + TENS) vs. (TLSO)	−0.17 ± 0.87 (−1.96 to 1.93)	0.98 (−0.11)
	T2	(TLSO + TENS) vs. (TLSO)	−0.5 ± 0.83 (−2.35 to 1.35)	0.56 (−0.35)
	T3	(TLSO + TENS) vs. (TLSO)	−1.63 ± 0.70 (−3.20 to −0.07)	0.04 (−1.34)
Ti/Ttot	T1	(TLSO + TENS) vs. (TLSO)	−0.01 ± 0.02 (−0.07 to 0.05)	0.69 (−0.29)
	T2	(TLSO + TENS) vs. (TLSO)	0.003 ± 0.02 (−0.04 to 0.04)	0.87 (0.09)
	T3	(TLSO + TENS) vs. (TLSO)	0.09 ± 0.03 (0.01 to 0.17)	0.03 (1.73)
**Pairwise comparisons of time points within each group**				
Outcome	Group	Conditions	MD ± SE (95%CI)	*p* value (Cohen’s d)
VAS (cm)	TLSO + TENS	T1 vs. T2	0.6 ± 0.13 (0.2 to 1)	0.004 * (1.88)
		T1 vs. T3	1.25 ± 0.22 (0.6 to 1.9)	0.001 * (2.32)
		T2 vs. T3	0.64 ± 0.15 (0.21 to 1.27)	0.004 * (1.74)
	TLSO	T1 vs. T2	0.36 ± 0.19 (−0.07 to 0.8)	0.09 (0.77)
		T1 vs. T3	0.44 ± 0.32 (−0.26 to 1.16)	0.19 (0.56)
		T2 vs. T3	0.08 ± 0.21 (−0.39 to 0.55)	0.71 (0.16)
TASS (total score)	TLSO + TENS	T1 vs. T2	1 ± 0.5 (−0.12 to 2.12)	0.07 (0.82)
		T1 vs. T3	0.00 ± 0.39 (−0.87 to 0.87)	1 (0.00)
		T2 vs. T3	−1 ±0.57 (−0.28 to 2.28)	0.11 (−0.72)
	TLSO	T1 vs. T2	1.66 ± 0.5 (0.54 to 2.79)	0.008 (1.36)
		T1 vs. T3	−0.33 ± 0.39 (−0.54 to 1.21)	0.41 (−0.35)
		T2 vs. T3	−2 ± 0.57 (−3.28 to −0.71)	0.006 (−1.43)
VT (mL)	TLSO + TENS	T1 vs. T2	11.5 ± 11.18 (−13.42 to 36.42)	0.32 (0.42)
		T1 vs. T3	−34.83 ± 6.43 (−49.16 to −20.50)	˂0.001 * (−2.21)
		T2 vs. T3	−46.33 ± 11.03 (−70.91 to −21.75)	0.002 * (−1.73)
	TLSO	T1 vs. T2	13.50 ± 11.18 (−11.42 to 38.42)	0.25 (0.49)
		T1 vs. T3	−18.5 ± 6.43 (−32.83 to −4.16)	0.01 (−1.17)
		T2 vs. T3	−32 ± 11.03 (−56.58 to −7.41)	0.01 (−1.18)
RR (breaths/min)	TLSO + TENS	T1 vs. T2	−0.84 ± 0.37 (−1.69 to 0.00)	0.05 (−0.93)
		T1 vs. T3	2.64 ± 0.82 (0.8 to 4.49)	0.01 (1.31)
		T2 vs. T3	3.49 ± 0.92 (1.44 to 5.54)	0.004 * (1.55)
	TLSO	T1 vs. T2	0.24 ± 0.37 (−0.6 to 1.08)	0.05 (0.26)
		T1 vs. T3	2.26 ± 0.82 (0.42 to 4.11)	0.02 (1.13)
		T2 vs. T3	2.02 ± 0.92 (−0.02 to 4.07)	0.05 (0.9)
Ti/Ttot	TLSO + TENS	T1 vs. T2	−0.02 ± 0.02 (−0.07 to 0.02)	0.22 (−0.41)
		T1 vs. T3	−0.12 ± 0.02 (−0.18 to −0.06)	0.001 * (−2.45)
		T2 vs. T3	−0.06 ± 0.01 (−0.12 to −0.06)	˂0.001 * (−2.45)
	TLSO	T1 vs. T2	−0.01 ± 0.02 (−0.06 to 0.03)	0.42 (−0.2)
		T1 vs. T3	−0.02 ± 0.02 (−0.08 to 0.03)	0.42 (−0.41)
		T2 vs. T3	−0.008 ± 0.01 (−0.04 to 0.02)	0.57 (−0.33)

Abbreviations: MD: Mean Difference; SE: Standard Error; CI: Confidence Interval; *p*: *p*-value; Cohen’s d: Effect size; VAS: Visual Analog Scale; TASS: Total Score; VT: Tidal Volume; RR: Respiratory Rate; Ti/Ttot: Inspiratory Time Fraction; TLSO: Thoracolumbosacral Orthosis; TENS: Transcutaneous Electrical Nerve Stimulation. * *p* < 0.006 was considered significant.

## Data Availability

The data supporting the findings of this study are available from the corresponding authors upon reasonable request. Due to privacy and ethical restrictions, the data are not publicly deposited.
